# Harveian Society of London

**Published:** 1889-05

**Authors:** 


### HARVEIAN SOCIETY OF LONDON.

*Meeting Held March 28, 1889.*

THE PRESIDENT, THOMAS BUZZARD, M. D., F. R. C. P., IN THE CHAIR.

### INGUINAL VERSUS LUMBAR COLOTOMY.

BY HARRISON CRIPPS, F. R. C. S.

The author, after giving an account of the history of colotomy, and drawing attention to the improvements in detail, introduced by Mr. Bryant and Mr. Davis Colley to the lumbar operation, further called attention to how much the profession was indebted to Mr. Reeves, Mr. Lawson Tait, Mr. Chavasse, Mr. Allingham and others for re-introducing the inguinal or intra-abdominal method of opening the bowel. After quoting Erckelen's and Batts' statistics, published in 1884, of several hundred cases of colotomy, collected from various sources, with a mortality of between 40 and 50 per cent., showed that such statistics were entirely misleading, and how with improved methods and hygienic surroundings the mortality might be immensely reduced. Of Mr. Harrison Cripps' own cases 15 were performed in the lumbar region, and 22 in the inguinal, nearly all the cases being undertaken for malignant disease. There was but a single death in each series of cases, thus the mortality was only a trifle over 5 per cent. Mr. Harrison Cripps, after thus vindicating colotomy as a fairly safe operation, proceeded to compare the inguinal with the lumbar method, stating that he considered the former vastly superior.

There were certain grave objections to the lumbar operation, amongst these were enumerated the depth of the bowel in a fat subject, the very limited space in which the surgeon had to work between the crests of the ilium and the last rib, which made it often difficult to find the bowel without severe damage to the surrounding tissues. Then, again, there was often a difficulty in recognizing the colon, so that numerous mistakes had been made in opening the small intestine, and even the stomach. But perhaps the gravest objection of all was that it not infrequently occurred that the course of the colon was so abnormal as to make it quite impossible to find it by the lumbar wound, the attempted operation ending in a fiasco. On the other hand, the inguinal operation met all these objections. There was plenty of space, the bowel could be absolutely identified, there was no tension on the stitches, and little difficulty in finding an abnormal colon. Moreover, the inguinal method had one great advantage entirely of its own, by enabling the abdomen to be explored and the site of the obstruction to be verified before opening the bowel, so that the mistake of being below the lesion could not occur. This was illustrated by two cases given in the table.

The objections raised to the inguinal operation were subsequent prolapse of the bowel, and that it was not suitable for urgent cases. In the author's experience prolapse was not more frequent from the one opening than from the other, and by a little care in the inguinal operation it could to a great extent be avoided. As to urgent cases he had no hesitation in opening the bowel immediately, as was done in two instances narrated with perfectly successful result. Mr. Harrison Cripps then described in some detail the operation he performed for inguinal colotomy. The incision, two inches and a-half in length crossed an imaginary line drawn from the anterior-superior spine to the umbilicus an inch and a-half from the former bony part.

In making the incision the skin should be drawn a little inwards, so as to make the opening somewhat valvular. The peritoneum being reached, it is pinched up by fine forceps, and an opening made sufficient to admit the finger. The intestines being protected by the finger, the peritoneum is divided by scissors to nearly the full length of the cutaneous incision. The colon may now at once show itself, and can easily be recognized by its longitudinal bands, its glandulæ epiploicæ, and by its regular convoluted surface. In about a third of my cases the large intestine presented at once; in the others either the small intestine, omentum, or mesentery first appeared. If any of these latter present, they must be pushed back and the colon sought for by the finger. Sometimes it can be detected by the hard scybalous masses within it, or it can be traced up after passing the finger into the pelvis and feeling for it as it crosses the brim.

The colon being found, a loop of it is drawn into the wound. In order to avoid the prolapse which is likely to occur if loose folds of the sigmoid flexure remain immediately above the opening, I gently draw out as much loose bowel as will readily come, passing it in again at the lower angle as it is drawn out from above. In this way, after passing through one's fingers an amount varying from one to several inches, no more will come. Two provisional ligatures of stout silk are passed through the longitudinal muscular band opposite the mesenteric attachment. These provisional ligatures, the ends of which are left long, help to steady the bowel during its subsequent stitching to the skin, and moreover, are useful as guides when the bowel is ultimately opened. They should be about two inches apart.

The bowel is now temporarily returned into the cavity. With a pair of fine forceps the parietal peritoneum is picked up, and attached to the skin on each side of the incision, the muscular coats of the abdominal wall not being included. Four sutures of fine Chinese silk are sufficient, two on each side, an inch and a half apart.

The bowel is again drawn out, and fixed to the skin and parietal peritoneum by seven or eight fine ligatures on each side, the last suture at each angle going across from one side to the other. The bowel should be so attached as to have two-thirds of its circumference external to the sutures. By turning the bowel slightly over, the lower longitudinal band can be clearly seen, and it is best to pass the sutures for the lower side through this, since it is a strong portion of the gut. The upper longitudinal band through which the provisional ligatures have already been passed is seen in the middle line of the wound. The bowel being now turned downwards, the opposite line of sutures are inserted close to its mesenteric attachment. No longitudinal band can, however, here be seen. The sutures, of the finest Chinese silk, are passed by small, partly curved needles, the needle passing through the skin one-eighth of an inch from the margin, then through the parietal layer of the peritoneum, and, lastly, partly through the muscular coat of the bowel, great care being taken to avoid perforating the mucous membrane. It is easier to pass all the threads before tying them up.

The wound should be most carefully and gently cleaned; the threads can then be all tied with moderate tightness. If the case is urgent, the bowel may now be opened; if not, a piece of green protective is put over it, a necessary precaution to prevent the granulations adhering to the gauze. The whole is covered with an antiseptic dressing, an additional thick pad being placed over the site of the wound. A broad flannel bandage is then wound firmly around the abdomen, so as to ensure considerable pressure. This is a most important precaution, for, should vomiting occur, the bowel is likely to burst away from the stitches. I also insist on the nurse sitting by the patient, with directions to press her hand firmly over the wound should sickness occur. When the patient becomes sensible, he can do this for himself. The wound is best dressed on the following day, to make sure that nothing has been misplaced.

If all goes well, the dressings may be reapplied, and the bowel not opened till the fifth or sixth day. The bowel being insensible, no anæsthetic is required. It will usually be found covered with a layer of lymph of surprising thickness. The provisional ligatures which have been left in will be found a useful guide, the bowel being opened to the full length between them. The superfluous flaps on either side are trimmed off with scissors to the level of the skin. In doing this one or two vessels require to be tied.

All ligatures may be safely removed by the ninth day, or earlier if there is redness around them. Firm pressure with a pad and bandage will be required for some time later.

He considered that it was a mistake to use too many sutures, this causing unnecessary strangulation of the skin, and preventing primary union. He saw no advantage in the suture passed through the mesentery, and had no difficulty in obtaining an excellent spur by attaching the bowel so that two-thirds of its circumference were above the level of the sutures.

The question as to when the bowel should be opened was considered as an important one. The author preferred to wait until the fifth or sixth day if all went well, but if the belly became distended, or there was the slightest vomiting, it should be opened at once. Firm pressure by a pad and several turns of bandage was very important for the first few days, for, with any strain on the part the bowel was liable to burst away.

The subsequent inconvenience arising from the new anus was but slight, the patient usually having a motion once a day with the knowledge of when it was coming, and power of controlling it by pressure.

In conclusion, Mr. Cripps stated that he considered the inguinal operation as now conducted was a marked advance in surgery, and he believed that before long the lumbar operation would be merely retained for a few exceptional cases of complete obstruction, and, moreover, now he trusted that it could be shown that the mortality was little more than 5 per cent., that surgeons would have more confidence in the operation, and would recognize its great value as a means of relieving suffering and threatened obstruction in advancing cases of malignant disease of the lower bowel.

In the discussion that followed,

Mr. Alfred Cooper said that during his twenty-five years' service as one of the surgeons to St. Mark's Hospital he had done and seen done many lumbar colotomies. He had ever felt most dissatisfied with the operation for the reasons mentioned by Mr. Cripps, and also for the difficulty experienced in maintaining cleanliness. Since Mr. Herbert Allingham's introduction of a modification of Littré's operation, Mr. Cooper had performed none but inguinal colotomies. He had found, however, that the omission of the deep suture had led to the passage of fæces into the lower gut.

Mr. Jacobson had of late been disappointed with inguinal colotomy. He had found a great amount of prolapse after the operation. He showed a patient, æt. 28, for whom he had performed lumbar colotomy just two years before. The artificial anus acted twice daily, and there was next to no prolapse. She was able to do household work. If inguinal colotomy is to become frequent, the lumbar operation, he thought, had still an eminent advantage for young married women. He believed also that the inguinal operation was undesirable in cases of sigmoid cancer, for it must here necessarily be performed near to the seat of disease. The frequency of cancerous disease of the sigmoid flexure was, he thought, under-estimated. His late colleague, Dr. Fagge, had shown that out of 100 cases of malignant disease of the large bowel, the seat of disease was in 4 cases the cæcum, in 10 cases the ascending colon, in 11 cases the transverse colon, and in 15 cases the descending colon, in 30 cases the sigmoid flexure, and in 30 cases the rectum. He still thought it a serious matter to open the peritoneum, as was necessary in performing the inguinal operation. The dangers of interfering with this membrane might be diminished, but never abolished, and that operation should be preferred which did not necessitate such a procedure. Mr. Jacobson wished to point out that when discussions such as this were published, operators less expert than those present were tempted to perform the operations described, upon patients in cottage hospitals and similar institutions, and he believed that to such operators the dangers of inguinal were greater than those of lumbar colotomy. Lastly, it must be remembered that the patient subjected to colotomy was often suffering from a mortal disease, and for such the gentler operation should be chosen. This he believed to be the extra-peritoneal operation. In performing the operation he believed it desirable to make a large opening, for the prolapse which might possibly follow was a lesser evil than the access of fæces to the lower portion of gut, such as occurred often when a small opening was made.

Mr. Swinford Edwards thought Mr. Jacobson's case a most favorable one. He had found prolapse quite as great in lumbar as in inguinal colotomy. He believed carcinoma of the sigmoid flexure to be very rare compared with that of the rectum. He was of the opinion that the advantages of inguinal over lumbar colotomy were four in number. It was easier of performance, the desired portion of the bowel was more easily found, the artificial anus was more conveniently placed for the patient, and also when there was considerable distention of the abdomen, the danger of turning the patient over was avoided. He had done 18 colotomies himself in the past two years. From this experience he thought it important to make a small abdominal incision to avoid prolapse of the bowel and of the abdominal contents. He considered the deep suture of importance as assisting in the formation of a spur. He believed it most important to cover the wound with green protective, as Mr. Cripps had advocated. It was also essential to give adequate support after the operation. The want of this in one of his cases had led to prolapse of the great omentum. He left this alone, and it gradually shrank away. He advocated inguinal colotomy as modified by Mr. Herbert Allingham in all cases requiring colotomy, even in those of acute obstruction, and he believed that extravasation of faces into the peritoneum could in all cases be prevented.

Dr. Ball, of Dublin, believed that Dr. Cripps had made out a good case for the intra-peritoneal against the extra-peritoneal operation of opening the colon. He further thought it impossible in such an operation to open the intestine below the stricture, as he had seen done in the lumbar operation. He believed that a modification of the operation might completely cut off the access of fæces to the cancerous portion of intestine. He thought, too, that the union of bowel to skin might be more easily brought about in front than behind on account of the thinness of the skin in the former situation. By the inguinal method, we ensured a shorter distance between the artificial anus and the cancer, and thus left a greater surface of intestine available for purposes of absorption. A contra-indication of inguinal colotomy was stated to be extensive meteorism, and this he believed to be a cause of great prolapse of bowel not easily replaced. In one case he had seen it necessary to incise the small intestine to ensure its return, and the operation was fatal. In extensive meteorism he preferred the lumbar operation. If the inguinal method be adopted, the abdominal cavity was most easily opened through the linea semilunaris. This he demonstrated from a frozen section. By this operation no muscular fibres were wounded and union was more easy. If the incision were too low the deep epigastric artery might be avoided by taking a line of incision not below the intersection of the linea semilunaris with the line joining the umbilicus with the middle of Poupart's ligament. If it could be ensured that no fæcal matter would escape into the peritoneum, he thought it much better to complete the operation in one stage. He described the method of operating he himself adopted to avoid extravasation of fæces. The bowel was thereby clamped twice before the sutures were passed. He had performed the operation thus eight times, and always with success. He considered it objectionable to perform the operation *à deux temps*, Sutures in the intestine tended to produce vomiting, and consequently the risk of this was doubled when the operation was performed in two stages.

Dr. Curtis, of New York, said that in America colotomy was preferred to resection of the rectum in all severe and extensive cases of cancer. The inguinal operation was almost entirely performed in preference to the lumbar operation. He himself had operated upon a patient with great meteorism, and had found that the incision did not fall over the centre of the sigmoid flexure, and this led to great difficulty in the operation. An opening into the general peritoneal cavity thus produced, had had to be filled up with iodoform gauze before the bowel was sutured.

Mr. Bryant had had only a limited experience of inguinal colotomy. He had done it only for congenital malformation. He had seen several cases after this operation where severe prolapse had troubled the patient. He had not seen such prolapse after lumbar colotomy. He thought that lumbar colotomy was not more objectionable to the patient than was the inguinal operation. Cleanliness was just as easily ensured. He had rarely seen difficulty arise from prolapse in any of the large number of cases of lumbar colotomy he had performed. Some amount of prolapse was necessary to avoid the passage of fæces into the lower bowel. He had seen prolapse of the lower part of the bowel after lumbar colotomy, but this was not very troublesome. He fully believed that inguinal colotomy was an easy operation, but he doubted if lumbar colotomy was after all so difficult as was believed; and if it were, this was a point to be overcome by the surgeon for the good of the patient. It was not the object of the surgeon to invent an operation so easy as to be performed by any one however inexperienced. He had not experienced much difficulty in finding the portion of the bowel he wished to open. He, therefore, still advocated lumbar colotomy in preference to the inguinal operation. He believed still that it increased the dangers of an operation to open the peritoneum. He insisted also upon the greater risk of cancer attacking the wound in inguinal than in lumbar colotomy. In case of failure of the first operation it was much casier and much less dangerous to change the side of operation if the lumbar operation were done, than if the inguinal operation were adopted.

Mr. Herbert Allingham had performed eighteen inguinal colotomies, and had found it much easier than lumbar colotomy. He still thought, however, that lumbar colotomy had been abused, and that the mistakes made in the operation were due to carelessness. In lumbar colotomy the peritoneum was opened a great deal more frequently than operators were aware of, and it was, he believed, better to open the peritoneum to find the large intestine surely than to grope for it in uncertainty. He described the method of inguinal colotomy he himself had adopted. He believed that inguinal colotomy was of no use unless a good spur was procured. He had observed great procidentia of the large intestine after lumbar colotomy. He had also seen after inguinal colotomy prolapse of the lower portion of the bowel. This, he had suggested in a paper read before the Medical Society in November, 1888, should be treated by removing the prolapsed bowel, and he had seen no evil results occur from the adoption of such a course.

Mr. Benton had seen a case in which the prolapsed gut would half fill a peck measure. He believed that prolapse was as frequent from the lower as from the upper portion of the bowel. He generally opened the bowel on the third day, and he treated distention by pricking with a grooved needle.

Mr. Cripps, in reply, said that he was inclined to think Dr. Ball's suggestions as to the operation valuable. He had had little trouble from prolapse, and such as there had been was from the upper portion of the bowel. He considered the question of cleanliness not a very important point. He thought inguinal colotomy the more preferable because it was easier than the lumbar operation, for the latter might not only be difficult, but even impossible. He believed it had been shown that it was not much, if at all, more dangerous to wound the peritoneum than any other structure of the body.—*The Medical Press*, April 10, 1889.

